# Mr. Parkinson's Letter on the Committee of Apothecaries

**Published:** 1813-02

**Authors:** James Parkinson

**Affiliations:** Hoxton Square


*To the Editors of the Medical and Physical Journal.*

GENTLEMEN,

THE circumstances under which I was nominated on the Committee, at the general meeting of the Apothecaries, assembled at the Crown and Anchor Tavern, on Nov. 26; and the knowledge that the resolution I then had the honor of proposing has been supposed to imply a want of confidence in the then existing Committee, demand the following explanatory observations.

When, at that meeting, it was proposed, as a resolution, "that the evils (complained of) had arisen from the want of a proper "*superintending body*," I was anxious to learn of whom that body was to be composed; feeling assured, that on the talents and respectability of that body, would, in a great measure, depend the promoting of that which I conceived ought to be our primary object—the preservation of the PUBLIC HEALTH. Having attended only once before, I supposed that the nature of this superintending body had already been determined on; I therefore requested to be informed to whom the interests of the public and of the profession were intended to be thus committed. The Chairman, in answer, candidly stated, that the plan was not at present so matured as to allow him to state of whom this body was to be composed.

On looking at the names of the existing Committee, it could not be supposed for a moment that they could be influenced by any improper motive. Their judgment might however be biassed; and, as I knew that many of those gentlemen stood high on the list of the society or company of Apothecaries, I confess that I did not think it impossible, that a partiality for that establishment might lead to the idea of forming the superintending body from the Court of Assistants of that Company—a measure which I feared would not sufficiently assist us in obtaining such great and valuable benefits for society, as the securing of a suitable education, and the improving and regulating of the practice of the Apothecaries; amongst whom so large a body of ignorant pretenders is known to exist. Under this impression, I proposed to the meeting the seventh resolution;* which was immediately adopted and the mover was appointed one of the Committee.

* Resolved, VII. That the Committee report their proceedings from time to time, and that a general meeting be again called, should a requisition for that purpose be signed and sent to the present Chairman by twelve subscribers.

Being

Being thus placed in the Committee, I should certainly have availed myself of this resolution, and have brought the consideration of any proposed partial measure before a general meeting. But the resolution, and the caution which dictated it, appear to have been alike unnecessary: and it is with pleasure I aver, that in no meeting for the promoting of any measures, embracing both the interests of the public at large and those of a distinct body, could more liberality and disinterestedness be found. In no one instance, I am convinced, have the Committee allowed any petty, local, or individual, consideration, to interfere with their care for the amelioration of the state of the profession in general; and equally anxious have they been not to prefer any measure promising professional advantage only, to one which they had reason to hope might prove of more public benefit. The preservation of the public health, the promoting of the fair and honorable interests of the profession, and the advancement of medical knowledge, are the objects which they have kept constantly in view whilst forming those regulations, which will soon be laid before the public.

Having also proposed the existing resolution for a subscription to defray the expenses of an application to Parliament, I must take the liberty to request of gentlemen, through this medium, that there may be no unnecessary delay in transmitting the subscriptions. The true friend to humanity and science will not wait to count upon the probability of success, in so laudable a cause; but will, at once, secure it, by the promptness with which he yields his assistance.

Whilst concluding, I must regret that I permit justice to yield to delicacy, by not dwelling on the great obligations the medical public owe to Mr. Burrows, our zealous and indefatigable Chairman. I am, Gentlemen,

*Hoxton Square,* Your's respectfully,

*Jan.* 17, 1813. JAMES PARKINSON.

